# Simultaneous nephrectomy and coronary artery bypass grafting through extended sternotomy

**DOI:** 10.1186/1749-8090-7-79

**Published:** 2012-08-30

**Authors:** Algimantas Budrikis, Mindaugas Jievaltas, Sami Al Assaad, Sarunas Kinduris

**Affiliations:** 1 Department of Cardiothoracic and Vascular Surgery, Medical Academy, Lithuanian University of Health Sciences, Eiveniu str. 2, 50009, Kaunas, Lithuania; 2 Clinic of Urology, Hospital of Lithuanian University of Health Sciences, Kaunas, Lithuania

**Keywords:** CAD, CABG, Renal carcinoma, Simultaneous operation

## Abstract

**Background:**

The advances in surgical techniques, resuscitation and anesthesiology support over the last years have allowed simultaneous thoracic and abdominal operations to be made for cancer and concomitant severe heart vessel disease relieving the patient from several diseases simultaneously and achieving long lasting remission or cure.

**Clinical case:**

A simultaneous nephrectomy and coronary artery bypass grafting procedure through extended sternotomy is reported. A 63-year-old man with severe coronary artery disease was found to have renal carcinoma.

**Diagnosis:**

Postoperative pathological investigation of the tumor revealed the presence of renal cell carcinoma pT3a N0 M0, G2. Coronarography revealed advanced three-vessel coronary artery disease.

**Treatment:**

We successfully performed a simultaneous curative surgery for renal carcinoma and coronary artery bypass graft surgery under cardiopulmonary bypass using a novel technique of extended sternotomy. Simultaneous surgery thus appears to be a beneficial and safe approach for the treatment of coronary artery disease and resectable renal cancer in carefully selected patients.

## Background

Cardiovascular and neoplastic diseases are the main causes of death in Europe [1]. In addition, the number of patients who have both critical coronary artery disease (CAD) and surgically resectable cancer concomitantly has been raising as the proportion of elderly in the general population increases [2]. The advances in surgical techniques, resuscitation and anesthesiology support over the last years have allowed for simultaneous thoracic and abdominal operations to be made for cancer and concomitant severe heart vessel disease [3-6] relieving the patient from several diseases simultaneously and achieving long lasting remission or cure. The surgical approach to patients who have both critical CAD and resectable cancer is controversial. Traditionally the surgical procedures have been staged with the cardiac surgery performed first followed by the general operation at a later date. However the aforementioned technique carries the risk of rapid cancer growth due to the suppression of the immune function related to the CPB surgery [2,7-9]. Several recommendations over the last 2 decades have favored combined operations over those in two settings in carefully selected patients [2,7,8,10,11]. In this paper we report the case that successfully underwent concomitant curative nephrectomy for renal cancer and CABG under CPB through a novel technique of extended sternotomy.

## Case presentation

**Figure 1:**
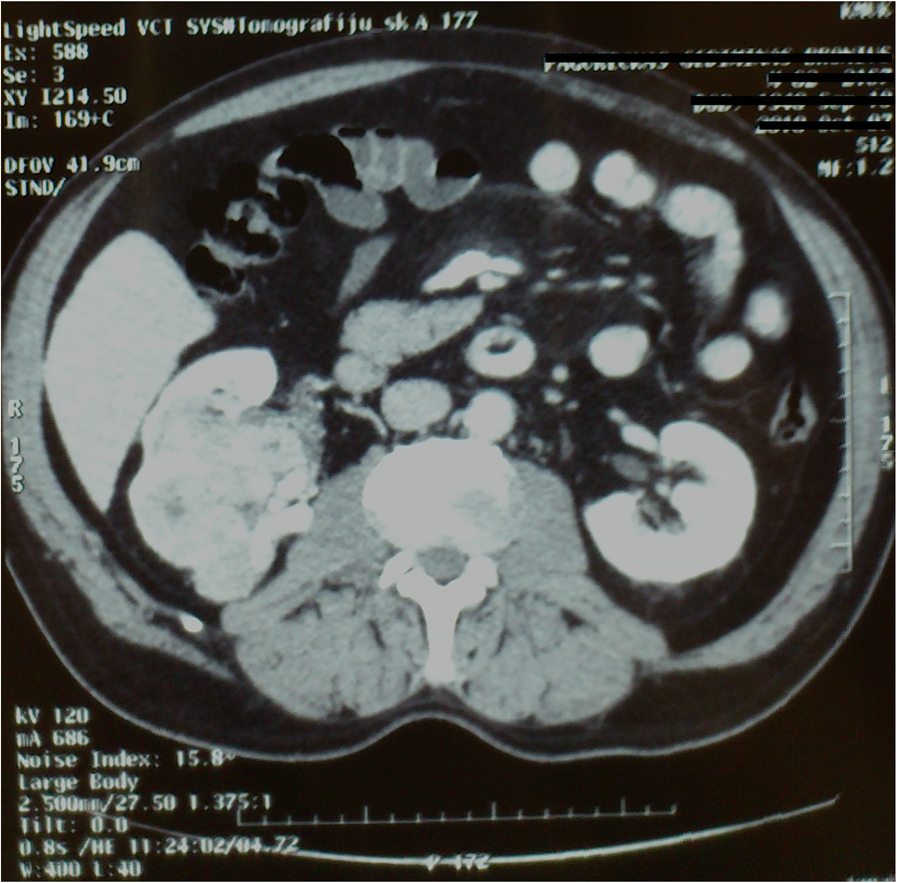
**Preoperative abdominal computed tomography scan.** The presence of renal tumor in the right kidney

**Figure 2:**
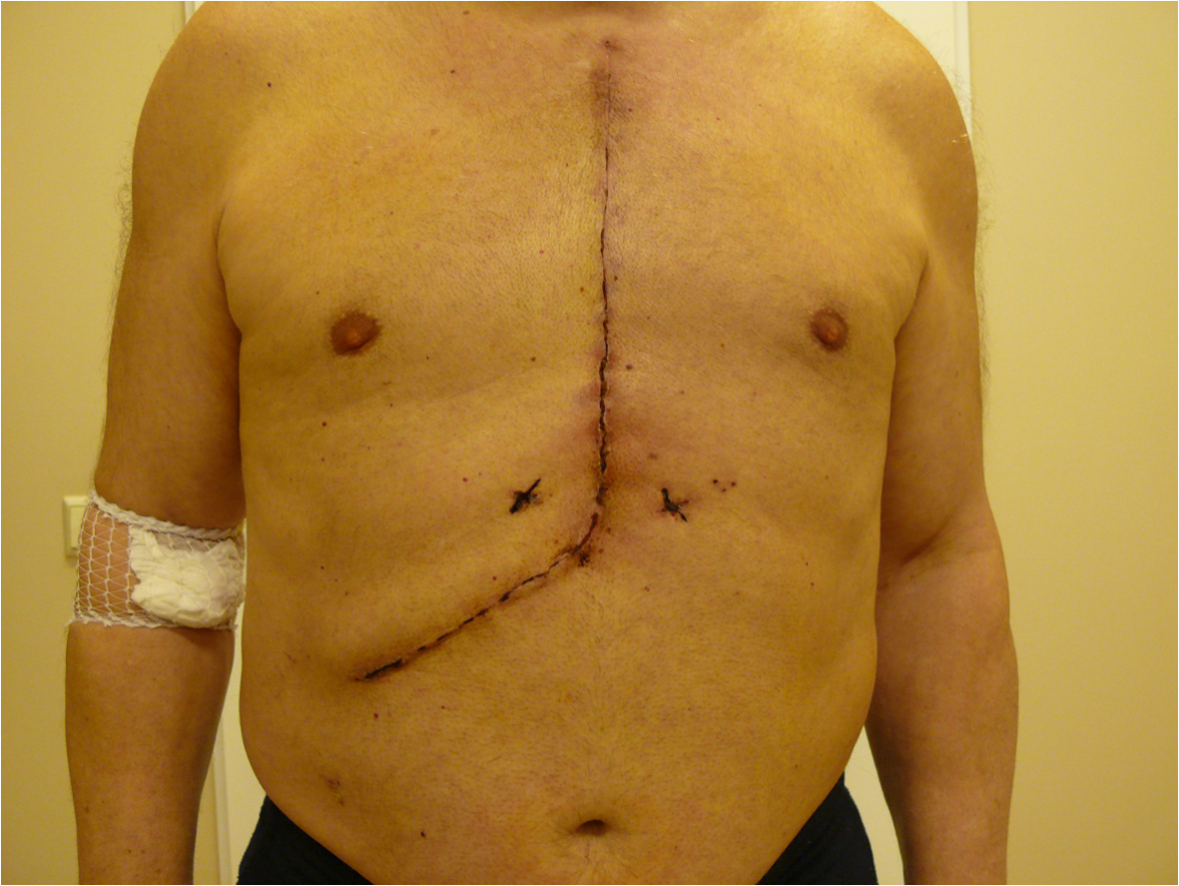
**Extended sternotomy.** The extended sternotomy incision post-operatively

A 63-year-old male patient was admitted to the department of cardiology because he had been suffering from recurrent anginal pain. The patient had a history of 2 MIs 10 years ago, as well as primary arterial hypertension for the last 20 years. The patient also had a history of hypertensive cardiomyopathy, and de novo renal carcinoma, which was diagnosed recently. Transthoracic echocardiography revealed good ejection fraction and no significant valve lesions. Coronary artery angiography showed stenoses in S2-50%; S4-75%; S11-75%; S12-75%; S13-90%; S6-65%; S8-75%; S10-100%. Abdominal CT revealed mixed polycyclic contours on the lower edge of the right kidney, with solid and cystic elements of a hypervascularized tumor of 10 cm (Figure 1). Deformation of the kidney contour was evident. A council was arranged in the presence of a cardiologist, a cardiac surgeon, an urologist and an oncologist where it was agreed that the patient would undergo a simultaneous operation of CABG under CPB and right nephrectomy. The kidney tumor had not metastasized. Decision to do concomitant operation was taken because of multiple coronary lesions and recurrent angina even during hospital stay. After the induction of anesthesia the surgical team started by making the incision, the technique used was the extended sternotomy, it involves making a median sternotomy and then continuing it through a right sub-costal incision (Figure 2). There were two major reasons for using this technique, first this enables the cardiac surgeon to intervene quickly in case the patient’s condition begins to deteriorate under the nephrectomy, second this technique avoids turning the patient around mid-way through the operation under general anesthesia to lay him on the back for sternotomy after having finished nephrectomy through a lateral incision. The retroperitoneum was accessed via Kocher technique, and the right kidney was removed. The size of the removed kidney was 10 × 5 × 9 cm, and the size of the removed tumor was 8 × 5 × 9 cm. The adrenal gland was left in place, no excessive hemorrhage was encountered, one drain was left in the abdominal cavity and the abdominal incision was closed in an effort to minimize wound exposure time and hence decrease the risk of infection. There was no difficulty in removing the kidney through this approach. CABG was performed under CPB. In total, six distal anastomoses were made. One of which was anastomosis of the left internal mammary artery (LIMA) to the left anterior descending (LAD) artery. The entire operation lasted 5 hr 50 min., of which 1 hr 50 min for nephrectomy and 4 hr for CABG. Postoperative pathological investigation of the tumor revealed the presence of renal cell carcinoma pT3a N0 M0, G2.The patient was discharged on the 7th post-operative day in good condition. One year postoperatively the patient underwent abdominal ultrasound and thoracic X-ray, both tests revealed no metastases.

## Discussion

Usually patients with cardiac disease and other co-morbidities have been managed in staged procedures [7]. However in recent years a new direction has been taken to combine operations when feasible. We must be aware of the increased operative risk for non-cardiac procedures performed on individuals with major CAD associated with myocardial damage and impaired ventricular functions as documented by Foster et al. [12]. Postponed tumor resection may amplify the risk of exposure to the immunosuppressive effects of CPB, which may have a harmful effect on tumor growth and spreading [2,7-9,13], especially when patient requires prolonged postoperative care [14]. It is of great importance to mention that late complications of extracorporeal circulation contribute indirectly to the expansion of the existing malignancy according to the international bibliography [15-17]. Additionally the doubling of costs has to be considered, as well as the advantage in avoiding a second procedure. In the last two decades abundant literature has favoring the results of simultaneous operations involving CAD and cancers [1,2,7,8,11,18]. The early and mid-term outcomes of combined neoplastic resection and cardiac operation have demonstrated that this approach is both feasible and safe in carefully selected patients [18]. Litmathe et al. [7] presented a series of six patients who had undergone combined procedures using extracorporeal circulation and urologic tumor resection. Four of which had undergone tumor nephrectomy and CABG simultaneously, whereas the other two had undergone tumor nephrectomy and aortic valve replacement (AVR). All six patients demonstrated satisfactory long-term survival. Rao and colleagues analyzed 30 patients who underwent simultaneous lung resection and cardiac surgery and demonstrated that CPB did not have detrimental effect on 5-year survival [17]. It is important to differentiate cases of combined CAD and lung cancer and those with gastrointestinal tract (GIT) malignancy. In GIT malignancy two cavities are opened and that is case of more frequent contamination and infection. Our results are in agreement with the latter as well as many other studies from the literature demonstrating similar successful operations and good prognosis. Recent studies have demonstrated the favorability of OPCAB over CABG under CPB in simultaneous operations [8,11], however CPB has been shown to affect neutrophils and platelets and results in complement activation, which may be beneficial in patients with malignancies [18]. From the many clinical investigations published, it appears clear that patients undergoing OPCAB tend to receive a lesser number of bypass grafts [19]. Thus due to the high number of grafts required by our patient, it was ultimately decided that CABG under CPB is a safer approach. According to the published literature, surgeons usually employ variations of a sub-costal approach or a median laparotomy to extract the kidney in combined nephrectomy and cardiac procedures [7]. The technique of extended sternotomy allows for easier manipulation of the surgical field as well as quick access to the heart in case complications arise. We also believe that performing the operation through the extended sternotomy technique promises early somatic and social rehabilitation and yields a better cosmetic effect, as well as theoretically decreasing the possible spread of the cancer. Certain disadvantage such as increased risk of bleeding due to systemic heparinization in a two cavity-operation has to be considered when planning simultaneous operations under CPB. Additionally the immediate perioperative load of quite a traumatic operation including several organ systems could be serious and impair the outcome [7]. Nevertheless simultaneous treatment of both diseases has several advantages: it decreases general anesthesia related risks such as pneumonia or drug-induced complications; prevents both neoplastic disease progression and risk of MI, decreases the risk of post-cardio-surgical bleeding after intra-operative anticoagulation by early removing of the tumor and reduces the two stage surgical stress of the patient [20]. In accordance with the majority of the data published in the literature, combined procedures did not negatively influence hospital morbidity and mortality. Simultaneous operations eliminate the necessity of a second operation and do not delay the postoperative oncological therapy. Long-term results are primarily determined by histological diagnosis and by the extent of the tumor [21,22].

## Conclusion

In our case a simultaneous nephrectomy and CABG operation was a good option for the removal of a renal tumor in a safe manner regarding the risk of myocardial infarction. Our results confirm the advantages of simultaneous operations over traditional staged operations, in patients where such an intervention is feasible. The use of CABG under CPB vs. OPCAB remains an individual choice per surgeon taking into consideration the condition of each patient; however the high number of grafts for this particular patient and the proven long term patency of the grafts after CABG under CPB ultimately warranted the use of the latter technique.

## Consent

Written informed consent was obtained from the patient for publication of this Case report and any accompanying images. A copy of the written consent is available for review by the Editor-in-Chief of this journal.

## Abbreviations

AVR: Aortic valve replacement; CABG: Coronary artery bypass graft; CAD: Coronary artery disease; CPB: Cardiopulmonary bypass; GIT: Gastrointestinal tract; LAD: Left anterior descending; LIMA: Left internal mammary artery; MI: Myocardial infarction; OPCAB: Off-pump coronary artery bypass.

## Competing interest

The authors declare that they have no competing interests.

## Author’s contributions

AB is cardiac surgeon; he was performing CABG operation and postoperative follow-up of the patient and reviewing the literature and writing an article. MJ is urologist. He was performing nephrectomy, consulting our patient regarding urology and oncology. SA is a member of our surgery team. He was reviewing literature, collecting data and writing an article. SK is a member of our surgical team. He was reviewing literature and drafting an article. All authors read and approved the final manuscript.

## Disclosure

The authors do not receive honoraria, consultation fees or support from cited companies.
